# Effectiveness of dapagliflozin as an insulin adjunct in type 1 diabetes: a semi-mechanistic exposure-response model

**DOI:** 10.3389/fphar.2023.1229255

**Published:** 2023-10-25

**Authors:** Victor Sokolov, Tatiana Yakovleva, Robert C. Penland, David W. Boulton, Weifeng Tang

**Affiliations:** ^1^ M&S Decisions LLC, Moscow, Russia; ^2^ STU “Sirius”, Sochi, Russia; ^3^ Clinical Pharmacology and Quantitative Pharmacology, Clinical Pharmacology and Safety Sciences, R&D, AstraZeneca, Waltham, MA, United States; ^4^ Clinical Pharmacology and Quantitative Pharmacology, Clinical Pharmacology and Safety Sciences, R&D, AstraZeneca, Gaithersburg, MD, United States

**Keywords:** continuous glucose monitoring, dapagliflozin, insulin, exposure-response model, SGLT-2 inhibitor, HbA1c, type 1 diabetes

## Abstract

**Introduction:** Dapagliflozin-induced improvement of glycemic control in patients with inadequately controlled type 1 diabetes (T1D) is complicated by the delicate balance between blood glucose and exogenous insulin. In this work, we developed a semi-mechanistic population exposure-response model using pooled patient-level data to characterize the joint effect of dapagliflozin and insulin on average daily glucose concentrations and glycated hemoglobin (HbA1c) levels in patients with T1D.

**Methods:** A non-linear mixed-effects model was developed in Monolix (Lixoft, France) and R software (R Project, www.r-project.org) using pooled patient-level data from phase 2 and phase 3 trials (NCT01498185, NCT02460978, NCT02268214).

**Results:** Because of the apparent lack of association between bolus insulin dose and glucose concentrations measured by continuous glucose monitoring the model was able to capture the quantitative link between basal, but not bolus, insulin dose and plasma glucose. Even so, this association remained flat, with a 50% decrease in the basal insulin dose from pretreatment level, resulting in ∼5% increase in glucose exposure. Therefore, dapagliflozin efficacy was not significantly affected by the insulin dose adjustment, with 24-week HbA1c reduction on 10-mg dapagliflozin treatment changing from −0.5 [95% CI: −0.55, −0.45] to −0.42 [95%CI: −0.48, −0.36] after adjustment. At the same time, the analysis revealed ∼2-fold steeper slope of glucose-HbA1c relationship in dapagliflozin-treated patients vs. control group, suggesting the presence of additional dapagliflozin treatment–related benefits, not explained by the dapagliflozin-mediated ∼4% increase in plasma hemoglobin levels. Finally, the efficacy of 5 and 10-mg doses, represented by the mean HbA1c reduction at week 24 of dapagliflozin treatment, was shown to be notably greater than the 1- and 2.5-mg doses.

**Discussion:** This research is an attempt to deconvolute and reconstruct dapagliflozin-HbA1c dose-response relationship in T1D by accounting for the drug’s action on both daily insulin dose and plasma glucose on a subject-level. While the model is able to adequately capture the observed data, it also revealed that the variability in CGM is poorly approximated by the variability in insulin dose alone. Furthermore, the slope of CGM/HbA1c relationship may differ depending on the population and treatment scenarios. As such, a deeper dive into the physiological mechanisms is required to better quantify the intricate network of glycemic response under dapagliflozin treatment.

## 1 Introduction

Although exogenous insulin is essential for the treatment of type 1 diabetes (T1D), the association of insulin treatment with suboptimal glycemic control and an increased risk of weight gain and hypoglycemia (Paik and Blair, 2019) underscores the need for adjunctive treatments. Basal bolus insulin regimens are commonly used in individuals with T1D to emulate the physiological action of defunct pancreatic beta cells; basal insulin provides long-acting stable glucose control, whereas bolus insulin provides rapid-acting prandial glucose control (Vliebergh et al., 2021). Sodium-glucose co-transporter-2 (SGLT-2) inhibitors decrease plasma glucose levels in an insulin-independent manner *via* inhibition of renal glucose reabsorption (Paik and Blair, 2019). Dapagliflozin is a selective SGLT-2 inhibitor that is approved in Japan as an adjunct to insulin in patients with T1D (AstraZeneca, 2019). The Dapagliflozin Evaluation in Patients With Inadequately Controlled Type 1 Diabetes (DEPICT-1; NCT02268214) (Dandona et al., 2017; Dandona et al., 2018) and DEPICT-2 (NCT02460978) (Mathieu et al., 2018) phase 3, placebo-controlled clinical trials reported improved glycemic control as well as reductions in total daily insulin doses following treatment with dapagliflozin as an adjunct to insulin in patients with T1D, although with an increased risk of diabetic ketoacidosis (Jendle et al., 2021).

Previously, we developed an exposure-response model that successfully described the longitudinal glycated hemoglobin (HbA1c) data from DEPICT-1 and DEPICT-2 (Parkinson et al., 2019). The complex network of interactive mechanisms among dapagliflozin treatment, insulin dose titration, plasma glucose concentrations, and HbA1c levels in patients with T1D substantiates the need for further mechanistic model-based analyses. In the present work, we built upon the previous approach to better understand the relationship between the glycemic effects of dapagliflozin and insulin dose adjustments. Accordingly, we developed an exposure-response model to characterize the joint effect of dapagliflozin exposure and the changes in daily insulin dose on average daily plasma glucose and HbA1c levels.

## 2 Methods

### 2.1 Clinical data

The objectives of this analysis were to quantify the relationship between dapagliflozin exposure (steady-state 24-h area under the concentration curve [AUC]) and HbA1c through the assessment of the interaction of dapagliflozin treatment and the daily insulin dose and their joint effect on the average daily plasma glucose concentrations measured by continuous glucose monitoring (CGM) and, consequently, HbA1c levels. We developed a population exposure-response model using pooled patient-level pharmacokinetic and pharmacodynamic data from the NCT01498185 (Henry et al., 2015) and DEPICT-2 (NCT02460978) (Mathieu et al., 2018) dapagliflozin clinical trials and validated using DEPICT-1 (NCT02268214) (Dandona et al., 2017; Dandona et al., 2018) clinical trial data. All the trials were randomized, double-blind, placebo-controlled, parallel-group studies conducted in adults with T1D. NCT01498185 was a phase 2, dose-ranging (dapagliflozin: 1, 2.5, 5, or 10 mg daily), safety, and pharmacokinetic/pharmacodynamic study undertaken over 2 weeks (*N* = 70; only inpatient data from the first 7 days of treatment were used in the model development, which is described in Section 2.3). NCT02268214 (*N* = 833) and NCT02460978 (*N* = 813) were 24-week phase 3 studies with similar designs that investigated the safety and efficacy of dapagliflozin (5 or 10 mg once daily) as adjunctive therapy to adjustable insulin in patients with HbA1c levels of 7.5%–10.5% (58–91 mmol/mol) at randomization. The studies included 28-week extension periods, but only the first 24-week double-blind period was included in the present analysis. The insulin titration approaches for basal and bolus insulin doses were different between the phase 2 and phase 3 trials. Details on insulin adjustments are described in the Supplementary Material.

### 2.2 Ethics

The included clinical trials followed applicable regulatory requirements and were performed in accordance with the Declaration of Helsinki and the International Council for Harmonisation Good Clinical Practice guidelines. All the participants provided written informed consent.

### 2.3 Model structure and steps

**FIGURE 1:**
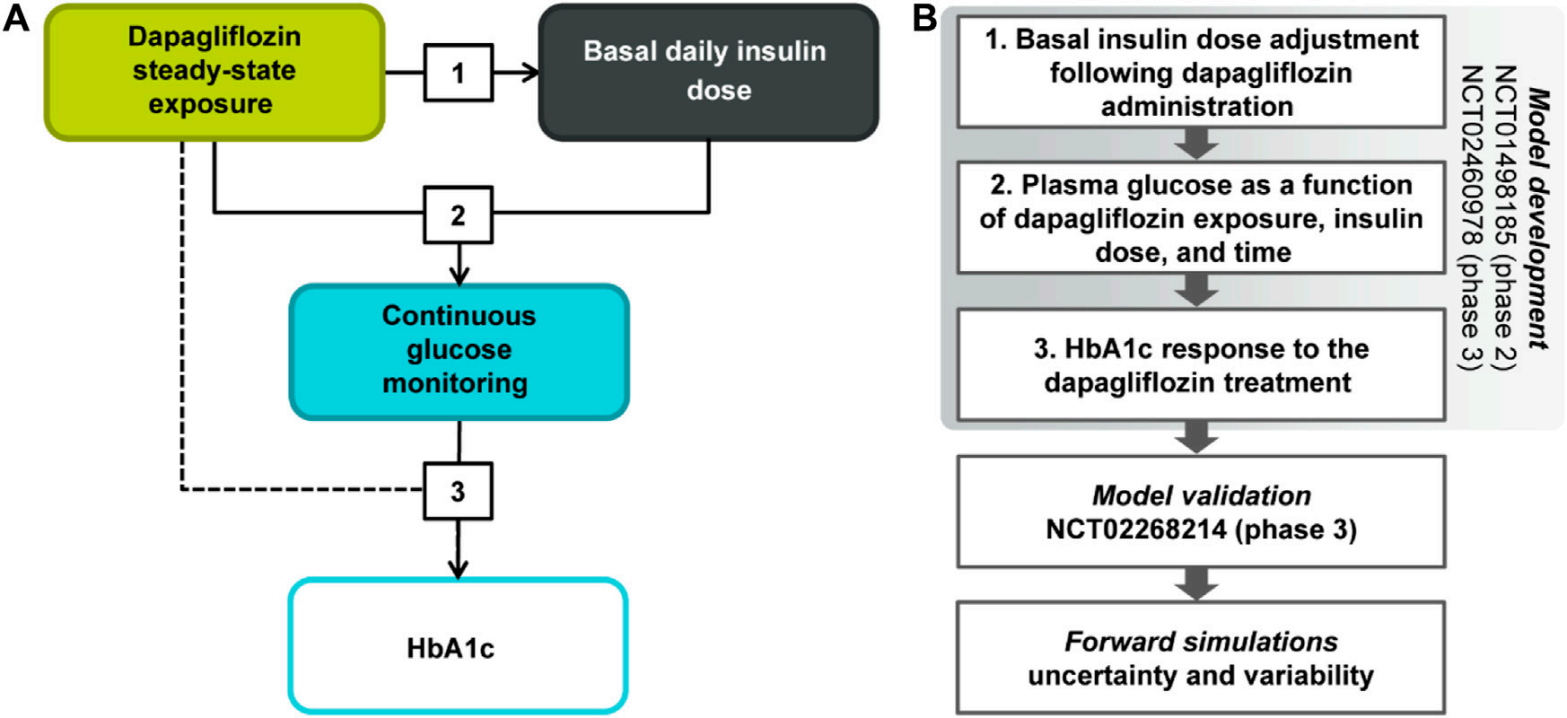
**(A)** Model scheme and **(B)** the analysis workflow. HbA1c, glycated hemoglobin.

A non-linear mixed-effects model characterizing the association between dapagliflozin steady-state 24-h AUC, change from baseline in insulin dose titration, plasma glucose concentrations, and HbA1c levels was developed using a stepwise approach (Figure 1). First, the reduction of basal insulin dose was parametrized as a function of dapagliflozin exposure following the exploratory analysis of the existing relationships between bolus and basal insulin doses and glucose. Next, the impact of basal insulin dose on average plasma glucose concentrations, the gradual increase of glucose with time in the absence of treatment, and the dapagliflozin-mediated glucose reduction were quantified. Finally, the relationship between average plasma glucose concentrations and HbA1c was established as the third step of model development.

At each stage of the modeling procedure, different statistical and structural models were evaluated in search of the optimal model. Discrimination between the models was based on changes in the objective function value, inspection of goodness of fit plots, identifiability of the parameters, and visual predictive checks. Further details of the model development and the final model parameters are provided in the Supplementary Material. The key measurements used in the analysis are shown in Table 1. Table 2 shows the number of patients and samples used in the modeling. Table 3 shows the final set of model parameters.

**TABLE 1:** Summary of measurements and measurement times.

Measurement	Unit	Measurement times (by study)
Dapagliflozin AUC	ng/mL*h	Taken from a previously performed population PK analysis (Melin et al., 2022)
Total daily bolus insulin dose	IU/mL	NCT01498185: 0–7 days
		NCT02268214, NCT02460978: 0, 2, 12, 24 weeks
Total daily basal insulin dose	IU/mL	NCT01498185: 0–7 days
		NCT02268214, NCT02460978: 0, 2, 12, 24 weeks
24-h mean of glucose from CGM, average per analysis visit	mg/dL	NCT01498185: 0–7 days
		NCT02268214, NCT02460978: 0, 12, 24 weeks
HbA1c	%	NCT01498185: no measurements
		NCT02268214, NCT02460978: 0, 4, 8, 12, 18, 24 weeks
Hemoglobin	g/L	NCT01498185: no measurements
		NCT02268214, NCT02460978: 0, 4, 8, 12, 18, 24 weeks

AUC, area under the concentration curve; CGM, continuous glucose monitoring; HbA1c, glycated hemoglobin.

**TABLE 2:** Number of patients and samples used in the modeling.

Dataset	Study	Number of patients	Number of measurements
Full dataset	NCT01498185 + NCT02460978	883	6,625
	NCT02268214	778	5,835
	Total	1,661	12,460
Step 1 Insulin dose vs AUC	NCT01498185 + NCT02460978	824	2,491
	NCT02268214	738	2,016
	Total	1,563	4,507
Step 2 CGM measurements vs AUC	NCT01498185 + NCT02460978	762	1,630
	NCT02268214	670	1,227
	Total	1,432	2,857
Step 3 HbA1c vs CGM measurements and AUC	NCT02460978	740	1,376
	NCT02268214	762	3,598
	Total	1,502	4,974

AUC, area under the concentration curve; CGM, continuous glucose monitoring; HbA1c, glycated hemoglobin.

**TABLE 3:** Summary of model parameters.

Modeling step	Parameter	Units	Description	Population value (omega)	RSE%
1	$Imax_{ins}$	–	maximum dapagliflozin impact on basal insulin dose	0.0941 (1.09)	8.14 (6.43)
	$IAUC50_{ins}$	ng/mL*h	the dapagliflozin exposure at which half of the maximum effect on insulin dose reduction is achieved	38.8	35.2
	$b_{ins}$	–	proportional residual error model parameter	0.185	1.93
2	k1	–	insulin impact on glucose	0.0674	29.7
	$k_{eff}$	mg/dL/week	time-dependent gradual increase in glucose	0.0015 (0.003)	21.5 (9.02)
	$Imax_{glu}$	–	maximum dapagliflozin impact on glucose	0.15	7.35
	$IAUC50_{glu}$	ng/mL*h	the dapagliflozin exposure at which half of the maximum effect on glucose is achieved	67.4	28.2
	$b_{glu}$	–	proportional residual error model parameter	0.162	1.99
3	k2	–	glucose impact on HbA1c	0.165 (0.739)	8.03 (8.08)
	$Imax_{hba1c}$	–	maximum dapagliflozin impact on HbA1c	0.0421	6.69
	$IAUC50_{hba1c}$	ng/mL*h	same as $IAUC50_{glu}$	67.4	–
	$a_{hba1c}$	–	constant residual error model parameter	0.061	2.15

AUC, area under the curve; HbA1c, glycated hemoglobin; RSE, relative standard error.

### 2.4 Software

Monolix software (version 2020R1) was used for non-linear mixed-effects modeling. Data visualization and forward simulations were performed using the R software version 4.0.2 (R-project, www.r-project.org).

## 3 Results

**FIGURE 2:**
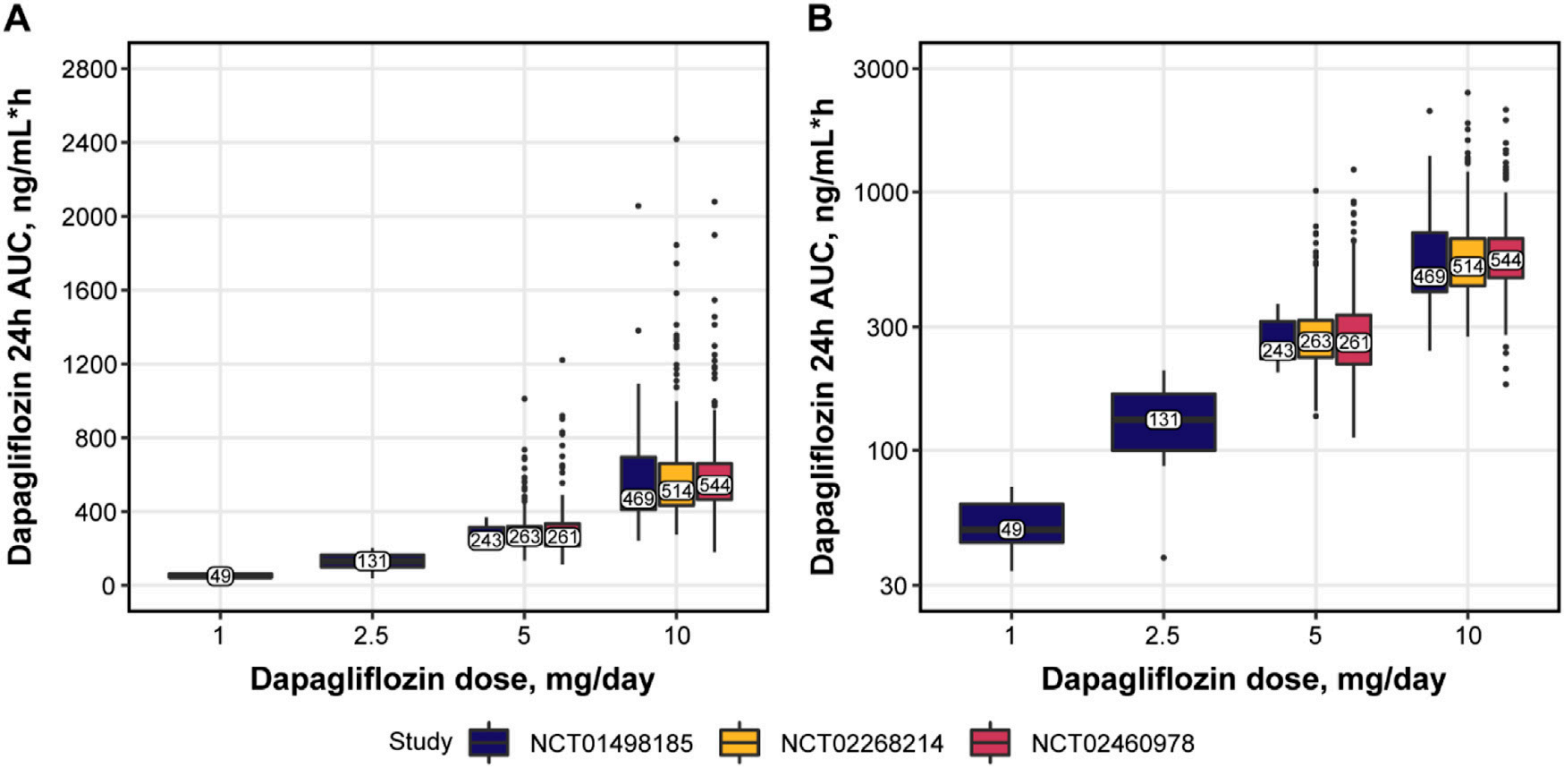
Dapagliflozin exposure in **(A)** normal and **(B)** log scale. Numbers and solid horizontal line = observed median; bars = IQR; whiskers = the range of values no greater than Q1—1.5 **×** IQR and Q3 + 1.5 **×** IQR; datapoints = values outside whiskers. 24h, 24 hours; AUC, area under the concentration curve; IQR, interquartile range; Q, quartile.

**FIGURE 3:**
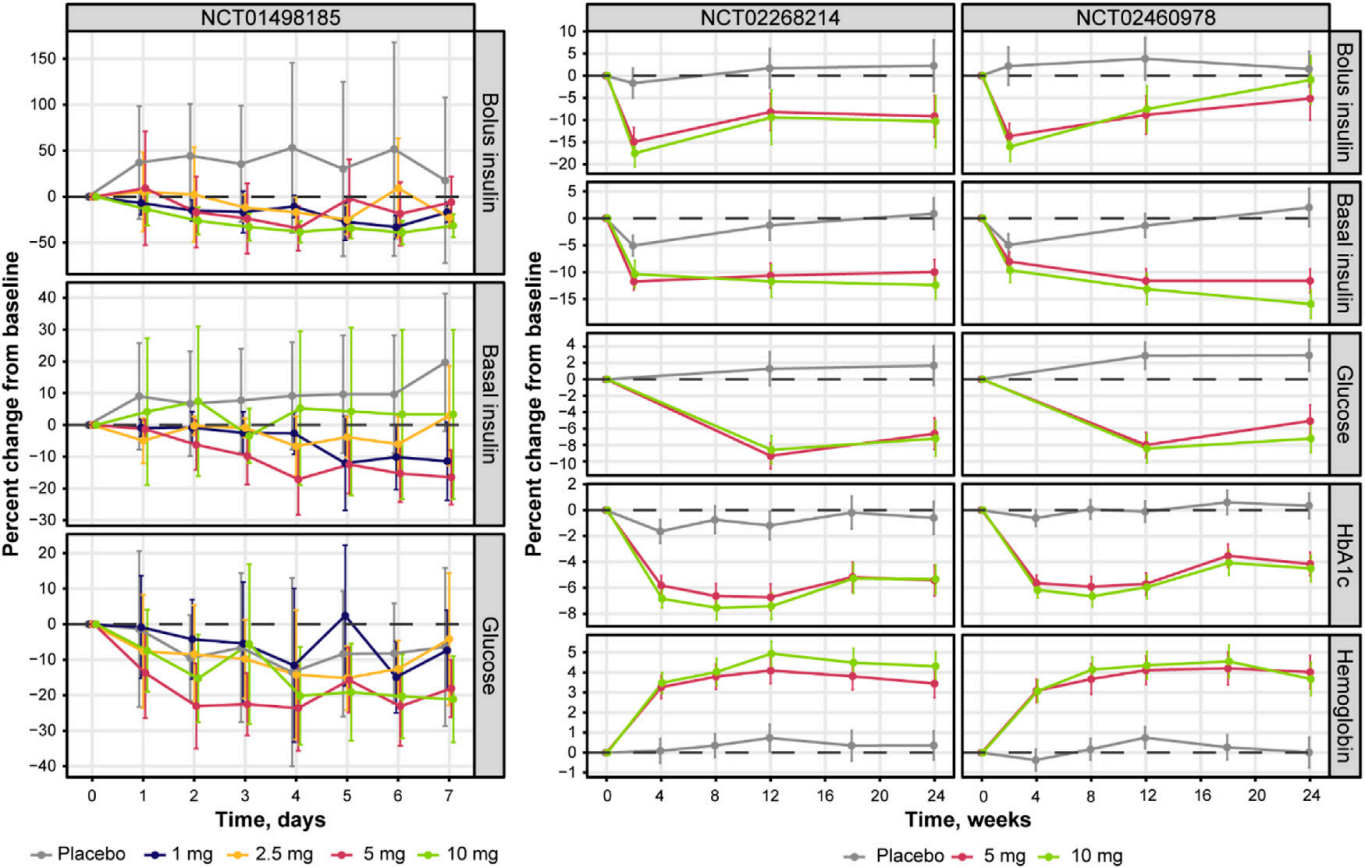
Percent change from baseline in bolus, basal daily insulin dose, plasma glucose concentrations, HbA1c levels, and plasma hemoglobin concentrations over time. Solid lines with points = observed means; whiskers = 95% CI of the mean. Glucose represents mean levels of plasma glucose concentrations, as assessed by continuous glucose monitoring. CI, confidence interval; HbA1c, glycated hemoglobin.

No major differences were observed in patient baseline characteristics between the studies (Table 4), except for lower median age and body mass index (BMI) in NCT01498185 and more Asian patients in NCT02460978. The overall increase in dapagliflozin exposure was dose proportional and comparable between the same dosing arms across the studies (Figure 2). Figure 3 shows the change over time in bolus and basal daily insulin doses, average glucose concentrations, HbA1c levels, and plasma hemoglobin concentrations. The major discrepancy between NCT01498185 and the phase 3 studies was the handling of the insulin dose in the placebo group. In NCT01498185, the daily bolus and basal insulin dose was increased by 40% and 10%, respectively, from baseline and remained stable throughout the inpatient period, whereas in the phase 3 studies, no changes were observed in the mean insulin dosing levels except for a 5% decrease in the basal insulin dose at week 2. Consequently, the placebo response in average glucose concentrations was comparable to that in the treatment groups for NCT01498185; in contrast, only a subtle increase in average glucose concentrations was observed in the placebo groups of the phase 3 studies. In all studies, dapagliflozin treatment resulted in a 10%–20% decrease in the daily insulin dose, both basal and bolus, on average. It could be noted that the change in the basal insulin dose was not dose proportional in NCT01498185, although the accuracy of this observation is challenged by variability within the data. The initial drop in basal insulin in all treatment groups of NCT02268214 and NCT02460978 studies was followed by a gradual increase in placebo-treated individuals, but not in dapagliflozin-treated individuals. In contrast, the bolus insulin dose in the 5-mg and 10-mg dapagliflozin groups increased over time in both DEPICT studies, whereas that in the placebo group remained stable. The average glucose concentrations decreased with dapagliflozin treatment by 10%–20% of baseline. Most of the reductions in HbA1c levels in the dapagliflozin treatment groups occurred over the first 4 weeks and remained stable, followed by a small increase at week 20. A 4% increase in hemoglobin concentration was observed under dapagliflozin treatment.

**TABLE 4:** Demographics and baseline characteristics of patients in the studies.

Characteristic	NCT01498185	NCT02268214	NCT02460978
Age, years	30 (18, 65)	43 (18, 75)	43 (18, 75)
BMI, kg/m^2^	23.9 (19, 33.4)	27.8 (18.2, 65.8)	26.9 (18.6, 56.6)
Body weight, kg	74.8 (51.9, 118)	80.8 (46.9, 184.8)	76.8 (44.6, 159.5)
CGM measurements, mg/dL	170.3 (106.5, 263.9)	189.5 (112, 302.4)	190.3 (65.1, 316.4)
Creatinine clearance, mL/min	120.9 (66.7, 282.6)	123.3 (47.5, 335.2)	117 (55.9, 289.2)
Diabetes duration, years	16.7 (2.5, 53.9)	18.7 (0.1, 65.8)	17.1 (0, 58.6)
eGFR, mL/min/1.72 m^2^	91.1 (49.9, 144.7)	89.4 (33.1, 175.3)	89.6 (26.1, 178.8)
FPG, mg/dL	145.8 (50.4, 295.2)	177.3 (30.6, 556.2)	176.8 (24, 472.5)
HbA1c, %	8.4 (7, 9.9)	8.4 (7.5, 10.4)	8.3 (7.5, 10.9)
Hemoglobin, g/L	139 (89, 163)	139.5 (97, 179)	139 (97, 178)
Total daily insulin dose, mU/L	48.3 (18, 165)	54 (1.9, 593.3)	51.5 (13.7, 208.6)
Sex (female)	30 (42.9)	405 (52.1)	455 (56.0)
Insulin: continuous subcutaneous insulin infusion	33 (47.1)	286 (36.8)	276 (33.9)
Insulin: multiple daily injections	37 (52.9)	492 (63.2)	537 (66.1)
Race			
** **American Indian or Alaska Native	─	11 (1.4)	1 (0.1)
** **Asian	1 (1.4)	1 (0.1)	160 (19.7)
** **Black or African American	5 (7.1)	15 (1.9)	12 (1.5)
** **Native Hawaiian/Other	1 (1.4)	─	1 (0.1)
** **Pacific Islander			
** **Other	1 (1.4)	7 (0.9)	2 (0.2)
** **White	62 (88.6)	744 (95.6)	637 (78.4)
Patients with concomitant treatment	52 (74.3)	653 (83.9)	707 (87.0)

Numbers are median (range) or n (%).

BMI, body mass index; CGM, continuous glucose monitoring; eGFR, estimated glomerular filtration rate; FPG, fasting plasma glucose; HbA1c, glycated hemoglobin.

**FIGURE 4:**
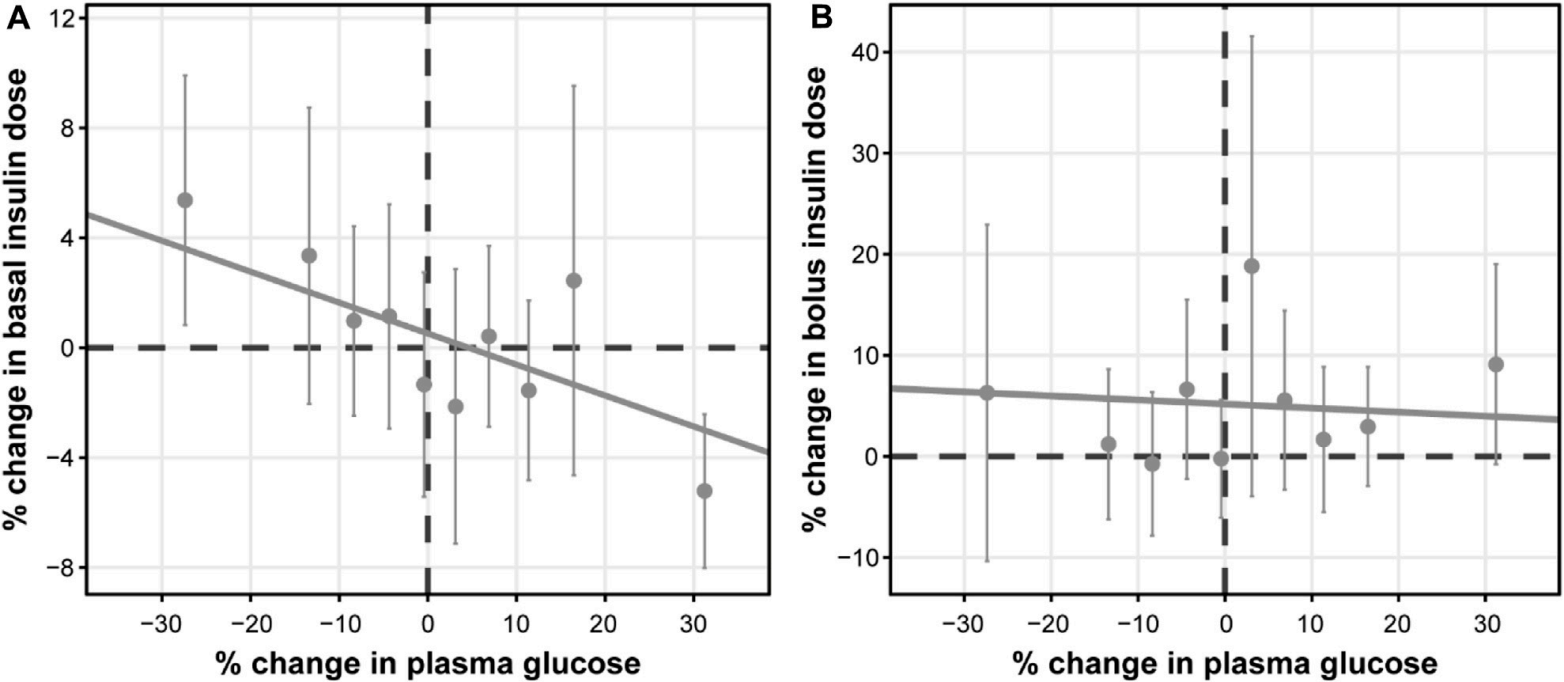
Correlation between the relative change from baseline in average plasma glucose concentrations and daily **(A)** basal, and **(B)** bolus insulin dose. Points with whiskers = observed mean with 95% CI; line = linear regression of the subject-level data; for **(A)** intercept = 0.516 (RSE: 140.16%) and slope = −0.113 (RSE: 39.29%); for **(B)** intercept = 5.19 (RSE: 35.35%) and slope = −0.04 (RSE: 280.26%). Plasma glucose concentrations represent mean levels, as assessed by continuous glucose monitoring. CI, confidence interval; RSE, relative standard error.

**FIGURE 5:**
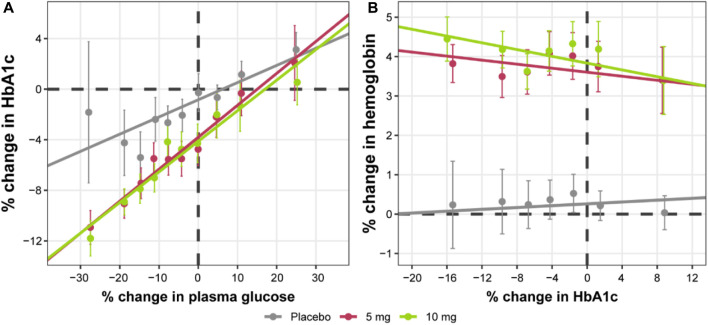
Correlation between relative change from baseline in HbA1c levels and **(A)** average plasma glucose concentrations or **(B)** hemoglobin levels. Points with whiskers = observed mean with 95% CI; line = linear regression of the subject-level data; for **(A)** placebo intercept = −0.839 (RSE: 29.41%) and slope = 0.136 (RSE: 11.86%); 5 mg intercept = −2.96 (RSE: 12.43%) and slope = 0.253 (RSE: 6.99%); 10 mg intercept = −3.28 (RSE: 11.3%) and slope = 0.242 (RSE: 7.12%); for **(B)** placebo intercept = 0.481 (RSE: 39.38%) and slope = −0.00191 (RSE: 646.74%); 5 mg intercept = 3.52 (RSE: 8.01%) and −0.000253 (RSE: 5329.06%); 10 mg intercept = 3.9 (RSE: 7.31%) and slope = 0.00133 (RSE: 989.5%). Plasma glucose concentrations represent mean levels, as assessed by continuous glucose monitoring. CI, confidence interval; HbA1c, glycated hemoglobin; RSE, relative standard error.

**FIGURE 6:**
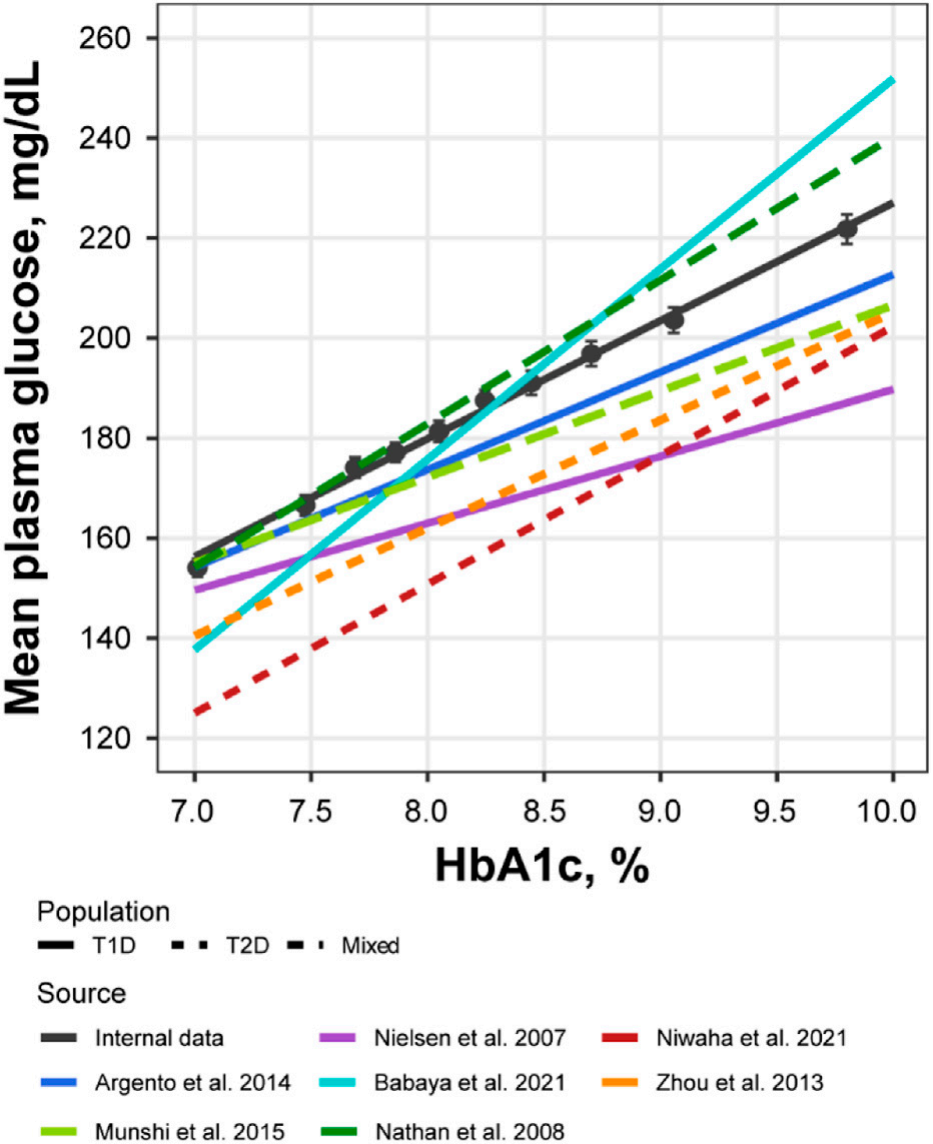
Correlation between mean plasma glucose and HbA1c levels compared across multiple sources. Black points with whiskers = observed mean with 95% CI; black line = linear regression of the subject-level data from NCT02460978 and NCT02268214 (Y = −8.84 + 23.6*X); colored lines = linear regression reproduced from the published sources: Y = 17.7 + 19.5*X for Argento et al. (Argento et al., 2014)., Y = 55.94 + 13.38*X for Nielsen et al. (Nielsen et al., 2007)., Y = −128.8 + 38.07*X for Babaya et al. (Babaya et al., 2021)., Y = −55.37 + 25.77*X for Niwaha et al. (Niwaha et al., 2022)., Y = −10.48 + 21.56*X for Zhou et al. (Zhou et al., 2013)., Y = 34.69 + 17.19*X for Munshi et al. (Munshi et al., 2015)., Y = −46.7 + 28.7*X for Nathan et al. (Nathan et al., 2007). CI, confidence interval; HbA1c, glycated hemoglobin; T1D, type 1 diabetes; T2D, type 2 diabetes.

Further exploratory analysis of the relative change in available measurements in placebo cohorts revealed the absence of an association between bolus insulin dose and CGM glucose measurements, whereas the correlation between plasma glucose and basal insulin dose was more noticeable: the slope for the linear regression of the former was −0.04 (relative standard error [RSE]: 280.26%) and −0.113 (RSE: 39.29%) for the latter (Figure 4). The most interesting observation, however, can be attributed to the differences in the slopes of the CGM glucose and HbA1c relationship between the control and active arms of the studies (Figure 5A). The slope for the dapagliflozin-treated groups of patients was approximately two times steeper than that of the control (0.136 [RSE: 11.86%] vs. 0.253 [RSE: 6.99%] and 0.242 [RSE: 7.12%]). This effect could be explained by the increase in the hemoglobin pool under dapagliflozin treatment. However, the absence of a correlation (RSE for the slopes in all subgroups >500%) between the changes in hemoglobin and HbA1c levels contradicts this hypothesis (Figure 5B). Further evaluation of the historical data revealed marked variability in the linear association between absolute concentrations of mean plasma glucose and HbA1c, with the intercept and the slope varying between −128.8 and 38.07 (Babaya et al., 2021), and 55.94 and 13.38 in (Nielsen et al., 2007), suggesting the existence of additional factors that influence this relationship (Figure 6).

**FIGURE 7:**
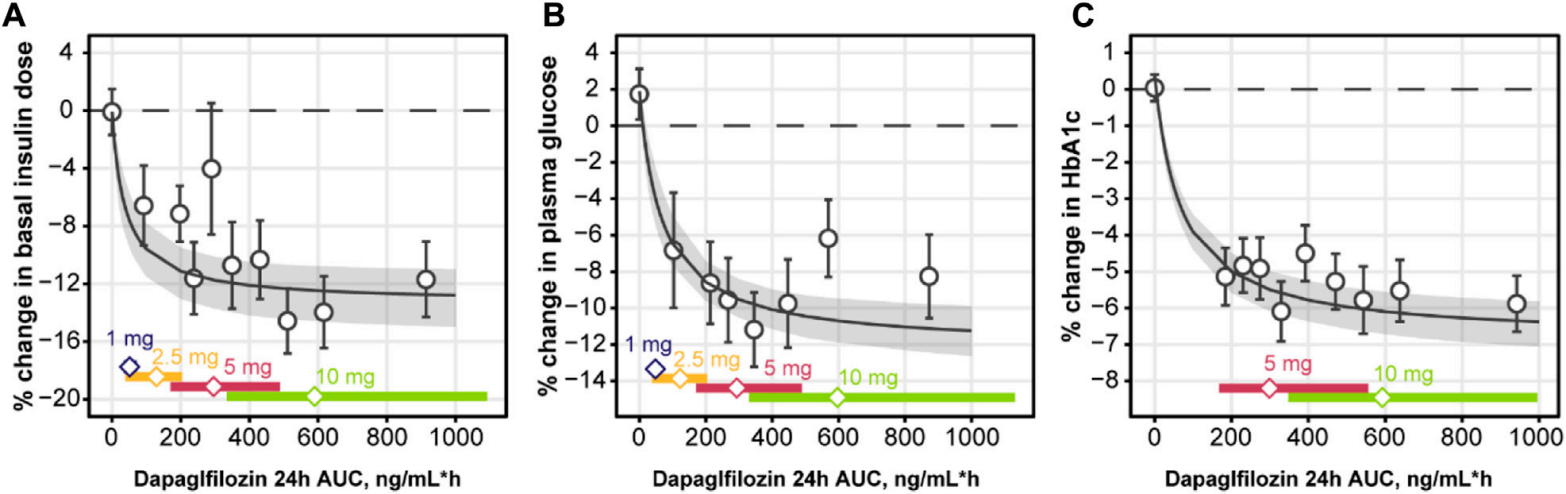
Observed and predicted change from baseline in basal insulin dose **(A)**, plasma glucose **(B)**, and HbA1c **(C)** over weeks 24 of daily dosing of dapagliflozin. Empty circles with whiskers = observed mean and 95% CI; solid line with shaded area = predicted population mean with 95% CI based on the simulation of 500 populations with 500 subjects and averaged across 24 weeks of treatment; empty diamonds = observed median dapagliflozin exposure for the selected dose; colored bars = the range between 2.5% and 97.5% percentile of the observed dapagliflozin exposure. CI, confidence interval; HbA1c, glycated hemoglobin.

**FIGURE 8:**
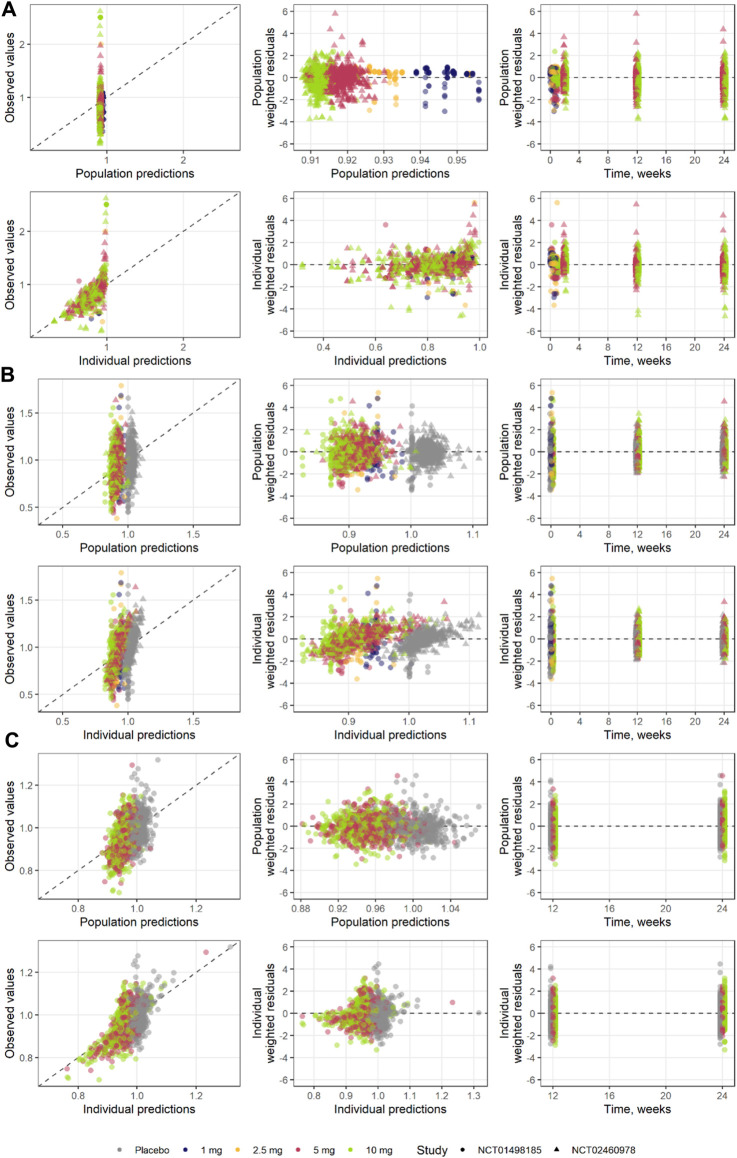
Goodness of fit for each step of the model development **(A)** step 1, **(B)** step 2, and **(C)** step 3.

Based on the observations listed above, a mathematical model was developed to capture the association between the four main markers of the analysis: daily dapagliflozin exposure, basal insulin dose, plasma glucose, and HbA1c (Figures 7, 8). The model described a negative association between basal insulin dose reduction and dapagliflozin concentration (Figure 7A). Although the glucose data were variable (placebo data standard deviation = 16.4%), the model successfully captured the intricate effect of insulin dose reduction and dapagliflozin treatment on plasma glucose together with the time-dependent function describing the treatment-independent trends in glucose dynamics that allowed the model to capture the mean increase in plasma glucose in placebo data. An increase in the plasma glucose over time is also the reason for the apparent underprediction of the average dapagliflozin-mediated benefit in Figure 7B for concentrations above 500 ng/mL*h, as phase 3 CGM measurements were available only at weeks 12 and 24 of treatment, when the placebo effect is more prominent compared to the first week of treatment in the NCT01498185 study. Finally, the association between dapagliflozin and HbA1c response was quantified (Figure 7C). As expected from the exploratory analysis, characterization of the association based only on the link between CGM measurements and HbA1c resulted in marked underprediction of HbA1c treatment response. The inclusion of hemoglobin as a factor influencing HbA1c within the model structure did not dissipate the apparent glucose-independent treatment effect. As such, to describe the data, an additional, direct effect of dapagliflozin on HbA1c was introduced.

**FIGURE 9:**
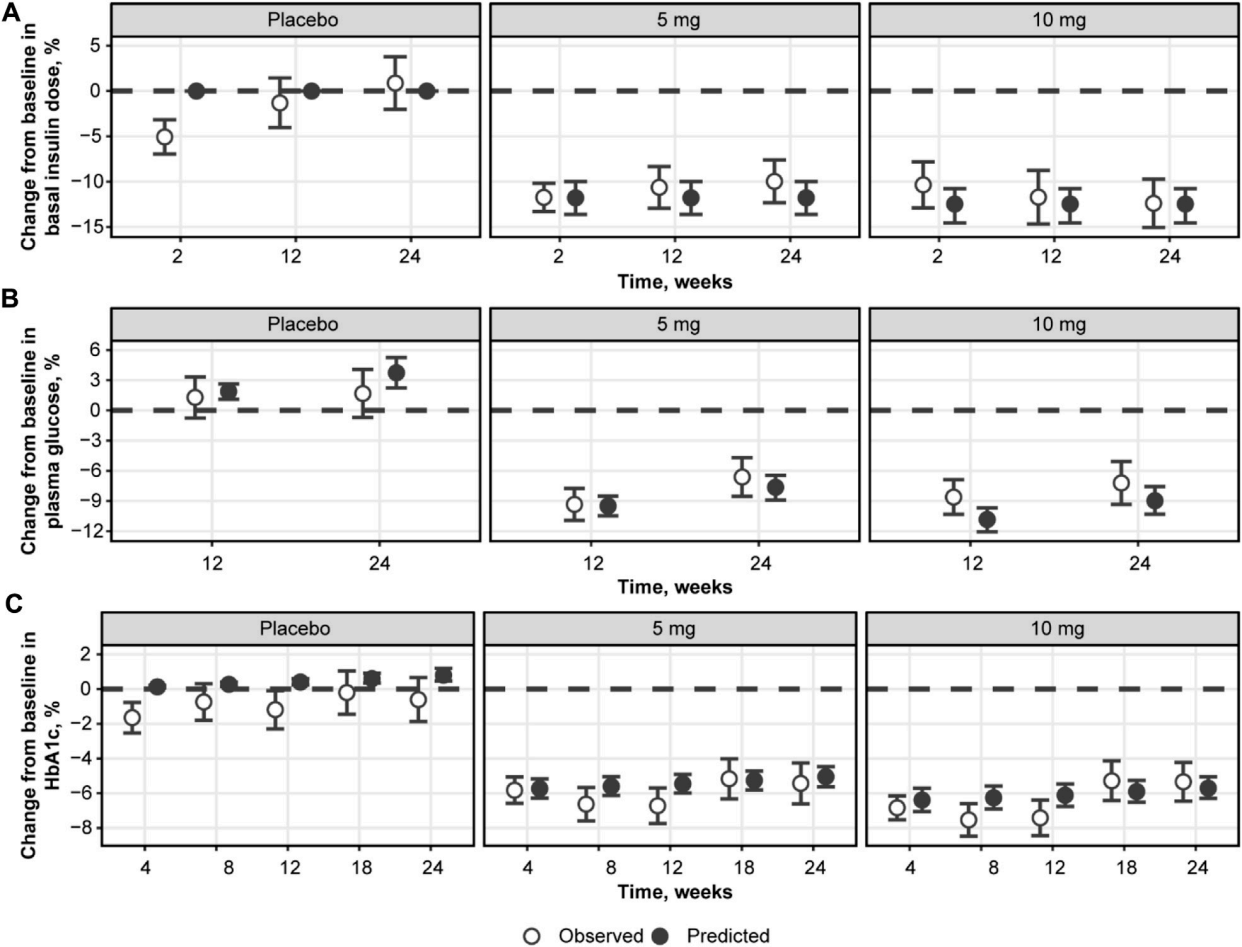
Model validation using NCT02268214 study data on the percent change from baseline in basal insulin dose **(A)**, plasma glucose **(B)**, and HbA1c **(C)**. Empty circles with whiskers = observed mean with 95% CI; solid circles = predicted population mean with 95% CI based on the simulation of 500 populations with 500 subjects. Glucose represents mean levels of plasma glucose concentrations, as assessed by continuous glucose monitoring. CI, confidence interval; HbA1c, glycated hemoglobin.

**FIGURE 10:**
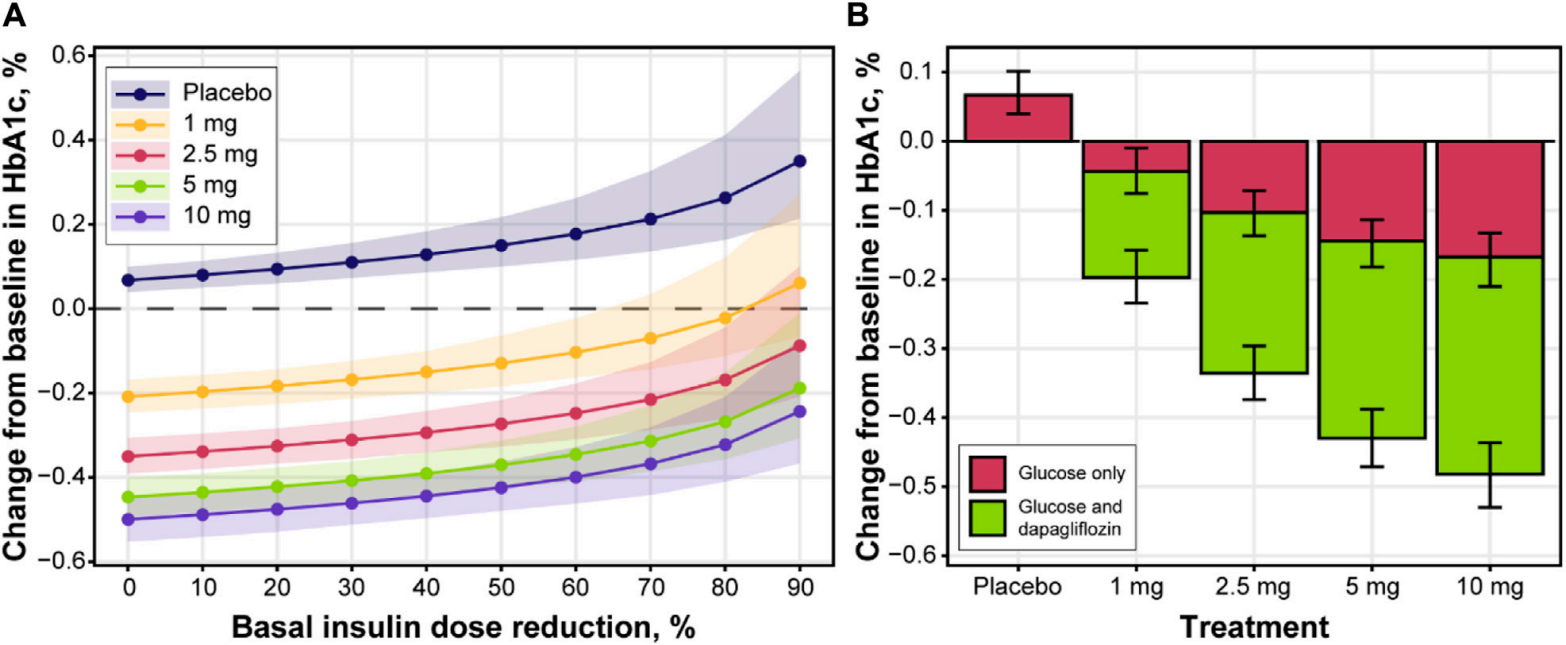
The impact of insulin dose titration on **(A)** HbA1c levels and **(B)** dapagliflozin dose-response at week 24. Lines with points and shaded area **(A)** or bars with whiskers **(B)** = predicted population mean with 95% CI based on the simulation of 500 populations with 500 subjects; color in **(B)** denotes the effect of glucose changes only on HbA1c (red color) or both dapagliflozin and glucose (green color); mean baseline HbA1c = 8.48%; mean AUC for 1, 2.5, 5 and 10 mg were 51.4, 130.6, 294.5 and 594.3 ng/mL*h, respectively. AUC, area under the curve; CI, confidence interval; HbA1c, glycated hemoglobin.

Model performance was assessed through independent validation using NCT02268214 study data, which were not used in model development (Figure 9). The model does not include mechanisms to describe a 5% decrease in basal insulin at week 2 of placebo treatment; however, the rest of the insulin dosing data were described well, along with CGM glucose measurements. Mean trends in the HbA1c profiles of the validation dataset were captured by the model, arguably with a small overprediction in the placebo response driven by the glucose increase over time. As such, the model performance was deemed satisfactory and the model itself suitable for subsequent forward simulations. First, to explore the degree to which basal insulin dose titration might influence the efficacy of dapagliflozin, we performed population simulations with a fixed insulin dose reduction followed by placebo or dapagliflozin treatment. Insulin dose reduction was varied over a wide range (0%–90% change from baseline) to analyze the model behavior (Figure 10A). As the glucose-insulin dose correlation was relatively flat, basal insulin dose reduction did not significantly affect the treatment-mediated benefits in HbA1c. The 24-week HbA1c reduction under 10 mg dapagliflozin treatment without insulin dose adjustment was −0.5% (95% CI: −0.553, −0.448), whereas a 50% reduction in the basal insulin dose resulted in a marginal 0.08% increase in the mean population response up to −0.42% (95% CI: −0.48, −0.36). For the 5- and 10-mg doses, the upper bound of 95% CI for the mean remained below zero, suggesting that the dapagliflozin treatment benefit might be observed even in extreme scenarios of basal insulin dose adjustment.

To further explore the efficacy of dapagliflozin at different doses, including the additional treatment effect parametrized by the model, mean population changes in HbA1c after 24 weeks of treatment were simulated under daily dapagliflozin or placebo administration and insulin dose adjustment driven by dapagliflozin exposure (Figure 10B). A linear increase in plasma glucose concentration over time was reflected in 0.07 (95% CI: 0.04, 0.1) increase in HbA1c levels in control arm of the simulation. A substantial contribution of the stand-alone dapagliflozin effect toward HbA1c reduction was observed at all doses. At 5 and 10 mg doses, this effect comprised approximately 65% of the total treatment benefit. According to the *in silico* results, dapagliflozin efficacy under 1 mg (−0.2 [95% CI: −0.23, −0.16]) and 2.5 mg (−0.34 [95% CI: −0.37, −0.3]) doses was inferior to the 5 mg (−0.43 [95% CI: −0.47, −0.39]) and 10 mg (−0.48 [95% CI: −0.53, −0.44]) treatment scenarios.

## 4 Discussion

Appropriate titration of insulin therapy is key to optimal glycemic control in patients with T1D. Reducing insulin doses in a controlled manner without losing the glycemic control is important to avoid the adverse effects of insulin treatment. Consequently, other glucose-lowering agents, such as SGLT-2 inhibitors, may be attractive as adjunctive therapies (Jendle et al., 2021). Understanding the interactions between insulin and dapagliflozin can facilitate their concomitant use. In this analysis, the semi-mechanistic exposure-response model we developed successfully established quantitative links among dapagliflozin exposure, the basal daily insulin dose, plasma glucose concentrations, and HbA1c levels in patients with T1D.

The model structure was selected based on the basic physiological facts of plasma glucose concentrations positively correlating with HbA1c, insulin stimulating glucose clearance from plasma to tissues, and dapagliflozin inhibiting renal glucose reabsorption (Nathan et al., 2007; Kasichayanula et al., 2014; Röder et al., 2016). A negative association between dapagliflozin and insulin dose was introduced to describe the insulin dose adjustment following additional glucose-modulating treatment. Two notable observations were made during the exploratory analysis of the available data. First, the correlation between plasma glucose concentrations measured by CGM and insulin dose was low in the case of basal insulin dose and absent in the case of bolus insulin (Figure 4). This highlights the limitation of the approach, as in reality, insulin dose adjustment is not driven by dapagliflozin exposure as an extrinsic factor, but by a combination of life activities (meals and exercise) as well as patients’ decisions, ultimately motivated by the plasma glucose measurements (Silver et al., 2018). As such, an increase in bolus insulin dose is most likely the result of compensatory behavior for high-sugar food intake and *vice versa* and does not correlate with mean daily glucose levels. The basal insulin dose is less susceptible to daily perturbations of glucose, and as a result, was included in the model.

Second, the linear association between average plasma glucose and HbA1c was not identical between the control group and treatment cohorts: more HbA1c reduction was observed for a fixed level of glucose concentrations in the dapagliflozin-treated patients compared to the placebo (Figure 5A). This is also emphasized by the lack of HbA1c increase in placebo cohorts throughout the course of phase 3 studies despite observing a 2%–4% increase in glucose concentration at weeks 12 and 24 on average (Figure 3). Consequently, the attempt to parametrize the relationship between average plasma glucose and HbA1c using a single set of parameters per patient resulted in either underprediction of placebo data or underprediction of treatment-mediated HbA1c reduction. Collectively, these results indicate the additional treatment-related benefits of dapagliflozin, with a significant contribution of approximately 65% to the total HbA1c response. One possible explanation for this observation is the “dilution” of HbA1c due to the increased rate of erythropoiesis observed with dapagliflozin. This explanation is consistent with a previous report showing a sustained increase in hematocrit in patients with type 2 diabetes treated with dapagliflozin who were also receiving chronic treatment with insulin (Aberle et al., 2020). However, our analysis of patient-level data did not show changes in hemoglobin concentrations that were directly associated with changes in HbA1c levels (Figure 5B). Another possibility to consider, although not demonstrated by the present data, is that weight loss mediated by dapagliflozin might decrease insulin resistance and increase the effect of the administered insulin.

Further analysis of the historical data revealed remarkable variability in the relationship between average plasma glucose and HbA1c levels (Figure 6) (Zhou et al., 2013; Argento et al., 2014; Henry et al., 2015; Munshi et al., 2015; Röder et al., 2016; Babaya et al., 2021; Niwaha et al., 2022). The potential reasons for such variety are many, including the short-term glycemic variability of which averaged characteristics, such as mean daily glucose and HbA1c, are not reflective (Munshi et al., 2015; Chehregosha et al., 2019). As such, another source of dapagliflozin efficacy might be the smoothing of daily glucose excursions, thereby sharpening the glucose-HbA1c relationship in dapagliflozin-treated patients. However, further studies are needed to understand the mechanisms underlying the additional effects of dapagliflozin on HbA1c reduction.

The developed mathematical model successfully captured the dapagliflozin-mediated reduction in insulin dose, plasma glucose, and HbA1c. By including both phase 2 and phase 3 data in the quantitative analysis, the model could be applied to predict glycemic responses to dapagliflozin treatment in a wider range of exposures, not only associated with the 5-mg and 10-mg doses used in the phase 3 DEPICT trials (Dandona et al., 2017; Dandona et al., 2018; Mathieu et al., 2018) but also with the 1 and 2.5-mg doses used in the phase 2 NCT01498185 trial (Henry et al., 2015). As expected, model-predicted HbA1c reductions under dapagliflozin treatment with 1 and 2.5-mg doses were lower than those with 5 and 10-mg doses. The ability to model the interactions between average daily glucose concentrations as measured by CGM and insulin dose could be very useful because it could guide clinicians in titrating insulin to maximize the benefit of dapagliflozin in patients with T1D. While our results are a step in this direction, in the current analysis, a poor correlation was found between average daily glucose concentrations as measured by CGM and insulin dose. The assumption that the insulin dose equals the insulin concentration in and across patients is most probably unfounded, and mean CGM data may not be the best description of systemic glucose exposure. Further evolution of the model toward a quantitative systems pharmacology–like analysis might be a valuable step for better understanding and simulating the short- and long-term responses to dapagliflozin treatment, with the goal of optimizing dosing regimens. Another semi-mechanistic exposure-response model (Perkins et al., 2020; Johnston et al., 2021) has been used to successfully predict the time-course and dose-related changes in HbA1c levels with SGLT-2 inhibitor treatment in patients with T1D, which boosts confidence in the current analysis. Drawing on modeling outputs, the authors predicted greater efficacy for patients receiving a stable insulin dose than for those in whom the insulin dose was adjusted when starting treatment with empagliflozin; they also confirmed the efficacy of low-dose (i.e., 2.5 mg) empagliflozin in patients with T1D (Perkins et al., 2020; Johnston et al., 2021).

In conclusion, a distinct benefit of the exposure-response model that we developed was the ability to integrate and help explain the complex and delicate relationships between dapagliflozin exposure, plasma glucose concentrations, insulin doses, and HbA1c levels within a single framework. Although each exposure response can be modeled separately, in reality, these parameters are not independent. The current model, therefore, provides an additional layer of complexity over existing models to quantitatively understand the intricate network of interactions in patients with T1D and provides a unique opportunity to better describe the efficacy of dapagliflozin in this population.

## Data Availability

The original contributions presented in the study are included in the article/Supplementary Materials, further inquiries can be directed to the corresponding author.

## Abbreviations

AUC, area under the curve; BMI, body mass index; CGM, continuous glucose monitoring; CI, confidence interval; eGFR, estimated glomerular filtration rate; FPG, fasting plasma glucose; HbA1c, glycated hemoglobin; RSE, relative standard error; SGLT-2, sodium-glucose co-transporter-2; T1D, type 1 diabetes.
