# Supplementary material for: Effectiveness of dapagliflozin as an insulin adjunct in type 1 diabetes: a semi-mechanistic exposure-response model
Source: Front Pharmacol. 2023 Oct 25;14:1229255. doi: 10.3389/fphar.2023.1229255 (PMC10634426; doi:10.3389/fphar.2023.1229255)
Supplement: Supplementary file 1 [file Table1.DOC]

Supplementary Material

Effectiveness of dapagliflozin as an insulin adjunct in type 1 diabetes: a semi-mechanistic, exposure-response model

**Victor Sokolov*, Tatiana Yakovleva, Robert C. Penland, David W. Boulton, Weifeng Tang**

***Correspondence:** Victor Sokolov: [victor.sokolov@msdecisions.ru](mailto:victor.sokolov@msdecisions.ru)

# Supplementary Data

**Basal and bolus insulin dose measurements and adjustments**

For NCT01498185, patients had to record the dose (in units) of all insulin taken (both basal and bolus) throughout each day. For NCT02268214 and NCT02460978, patients were instructed to record all their individual doses of basal and bolus insulin in their study diary at particular time periods (week −2 visit up to day 1 visit prior to randomization, day 1 visit after randomization up to week 2 visit, week 10 visit up to week 12 visit, and week 22 visit up to week 24 visit). At the particular time periods, patients were instructed to record the minimum and maximum total daily insulin dose range (basal plus bolus) taken for each week in their study diary (week −8 visit up to week −2 visit, week 2 visit up to week 10 visit, week 12 visit up to week 22 visit, and week 24 visit up to week 56 visit). For statistical analysis, the total daily dose was defined as the sum of all these individual doses of insulin (both basal and bolus) during a 24-hour period.

For NCT01498185, a standardized diet and guidance on insulin dose adjustments were provided to the patients. Although the insulin dose was not reduced at the start of treatment, investigators and patients were recommended to adjust the insulin dose for safety reasons and to minimize the risk of hypoglycemia. For the phase 3 NCT02268214 and NCT02460978 studies, the study investigators were recommended to reduce the daily insulin dose by up to 20% for both basal and bolus insulin after the first dose of study drug on day 1 to minimize the risk of hypoglycemia. Following any initial reduction in insulin dose, attempts were made to titrate insulin back to the baseline level, although insulin (mainly basal insulin) dose reductions were allowed before subsequent dapagliflozin administration. Insulin doses were adjusted at the investigator’s discretion based on self-monitored glucose readings and individual circumstances.

**Structural model**

A non-linear mixed-effects model was developed to characterize the association between dapagliflozin steady-state area under the concentration curve (AUC), basal insulin dose adjustment, plasma glucose, and glycated hemoglobin (HbA1c) in patients with type 1 diabetes.

In the first step of model development, the relative change from baseline (defined as the single available pre-treatment measurement per patient) daily basal insulin dose was described as a function of dapagliflozin exposure:

$$rINS_{ik}=\left( 1-\frac{Imax_{ins_{i}}*AUC_{i}}{IAUC{50}_{ins}+AUC_{i}} \right)$$

Equation 1

where $rINS_{ik}$ is the ratio to baseline in basal daily insulin dose measurement at k^th^ visit for the i^th^ patient, $AUC_{i}$ is the steady-state 24-hour dapagliflozin exposure in the i^th^ patient (ng/mL*h), $Imax_{ins_{i}}$ is the maximum dapagliflozin-mediated inhibitory effect on basal daily insulin dose in i^th^ patient, and $IAUC{50}_{ins}$ is the dapagliflozin exposure at which half of the maximum effect is achieved. $AUC_{ik}$ was obtained directly from the dataset as a regressor (time-varying covariate).

In the second step, dapagliflozin and insulin impact on the relative change from baseline in average daily plasma glucose concentrations, as measured by continuous glucose monitoring (CGM), was described using the following equation:

$${rGLU}_{ik}=\left( rINS_{ik} \right)^{k1}*\left( 1- \frac{Imax_{glu}*AUC_{i}}{IAUC{50}_{glu}+AUC_{i}} \right)+k_{eff_{i}}*TIME_{k}$$

Equation 2

where $r{GLU}_{ik}$ and $rINS_{ik}$ are the ratios to baseline glucose and basal daily insulin dose measurements at the k^th^ visit for the i^th^ patient, $TIME_{k}$ is the time of the k^th^ visit (weeks), $AUC_{i}$ is the steady-state 24‑hour dapagliflozin exposure in the i^th^ patient (ng/mL*h), $k1$ is a parameter that reflects the impact of insulin dose change on plasma glucose, $Imax_{glu}$ is the maximum dapagliflozin-mediated inhibitory effect on glucose, $IAUC{50}_{glu}$ is the dapagliflozin exposure at which half of the maximum effect on glucose clearance is achieved, $k_{eff_{i}}$ represents a linear treatment-independent change in glucose concentration over time in the i^th^ patient. For the parameter estimation procedure, $rINS_{ik}$ and $AUC_{i}$ were taken as regressors directly from the modeling dataset.

In the third and final step of model development, the quantitative link between change from baseline in plasma glucose and change from baseline in HbA1c was described by the following equation:

$$rHBA1C_{ik}=\left( {rGLU}_{ik} \right)^{k2_{i}}*\left( 1-\frac{Imax_{hba1c}*AUC_{i}}{IAUC{50}_{hba1c}+AUC_{i}} \right)$$

Equation 3

where $r{HBA1C}_{ik}$ and $r{GLU}_{ik}$ represent the ratio of baseline HbA1c and glucose measurements in the i^th^ patient at the k^th^ visit, with $k2$ reflecting the interplay between glucose and HbA1c in the i^th^ patient. $AUC_{i}$ is the steady-state 24-hour dapagliflozin exposure in the i^th^ patient (ng/mL*h), $Imax_{hba1c}$ is the maximum dapagliflozin-mediated inhibitory effect on HbA1c, and $IAUC{50}_{hba1c}$ is the dapagliflozin exposure at which half of the maximum effect on HbA1c is achieved. ${rGLU}_{ik}$ and $AUC_{i}$ were used as regressors for the parameter estimation procedure.

### Between-patient variability

Between-patient variability was introduced for either non-transformed parameter ($k_{eff}$) (**Equation 4**), log-transformed parameters such as $k2$ (**Equation 5**), or logit-transformed parameters, that is, $Imax_{ins}$ parameters (**Equation 6**):

$$P_{i}=P_{TV}+\eta_{i}$$

Equation 4

$${log(P}_{i})=\log\left( P_{TV} \right)+\eta_{i}$$

Equation 5

$$\log\left( \frac{P_{i}}{1-P_{i}} \right)=\log\left( \frac{P_{TV}}{1-P_{TV}} \right)+\eta_{i}$$

Equation 6

where $P_{i}$ is the value of $P$ for the i^th^ individual, $P_{TV}$ is the population typical value for the $P$ parameter, and $\eta_{i}$ represents a between-patient random effect that distinguishes the i^th^ individual’s true value from the population typical value; $\eta_{i}$ are independent and assumed to be normally distributed with a mean of 0 and a variance equal to ω^2^.

### *Residual variability*

Residual variability was modeled using one of the following error models:

$PRED=Y+a*\varepsilon$ for constant error model,

$PRED=Y+b*Y*\varepsilon$for proportional error model,

$PRED=Y+(a+b*Y)*\varepsilon$for combined error model type 1,

where $a$ and $b$ are the estimated error model parameters, $Y$ is the predicted value of a variable for a given patient at a given visit, $PRED$ is the value of $Y$ with residual error, and $\varepsilon$ is the normally distributed random variable with a mean of 0 and a variance of σ^2^.

### *Model discrimination*

Discrimination between models was mainly based on the inspection of graphical diagnostics and changes in the objective function value (OFV) provided by Monolix software. The difference in OFVs (ΔOFVs) is nominally χ2 distributed and a difference of −3.84 (larger model−smaller model) corresponds to approximately a *p* value of <0.05 for one degree of freedom, provided that the models are hierarchical. For a more complicated model to be retained, it had to provide a significant improvement over the contending model (*p* < 0.05 for nested models) and provide plausible parameter estimates not associated with excessively high relative standard errors (RSEs) (>50%). In the case of non‑nested models, Akaike Information Criterion (AIC) values were used to discriminate between models.

Model evaluation included graphical analysis of goodness-of-fit (GOF) plots, RSEs, and visual predictive checks (VPCs). Stratification (e.g., dose or covariates) was used when appropriate to ensure that the models perform adequately across important subgroups of the data. GOF plots included the standard set of dependent variables (DV) vs. population predictions (PRED), DV vs. individual predictions (IPRED), absolute individual weighted residuals (IWRES) vs. time, and absolute IWRES vs. IPRED.

**Model development**

In step 1, the effect of dapagliflozin exposure on the basal daily insulin dose was estimated using the data from the active arms of the studies by a model with proportional error and one random effect on logit-transformed $Imax_{ins}$ parameter (**Equation 1**).

In step 2, a model with single random effect on non-transformed $k_{eff}$ parameter and proportional error model was deemed suitable for further analysis (**Equation 2**). The model was used to estimate the impact of the basal daily insulin dose change on plasma glucose, dapagliflozin treatment-mediated response, and to characterize the gradual change in plasma glucose concentrations over time in the absence of dapagliflozin treatment, based on placebo and dapagliflozin treatment data for both phase 2 and phase 3 trials of the calibration dataset.

In the final step of the base model development, an attempt was made to parametrize the relationship between HbA1c and plasma glucose. However, based on the analysis of GOF and VPC plots, the parametrization of a model without additional components resulted in a consistent underprediction of the treatment effect (data not shown). To improve model predictions, an additional effect of dapagliflozin on HbA1c was tested in the structural model (**Equations 4**). However, the $IAUC{50}_{hba1c}$ parameter was not identifiable (RSE > 50%) and had to be fixed at the previously defined value of $IAUC{50}_{glu}$ = 67.4 ng/mL*h. The final model for step 3 included one random effect on the log‑transformed $k2$ parameter and constant residual error model.

### *Model validation and forward simulations*

After the model development procedure was finalized, the parameter estimates from each step of the modeling workflow were fixed at their optimal values. All subsequent forward simulations were performed using both the estimated variability in the form of random effects and uncertainty in the parameter values derived from the Fisher information matrix. For each simulation, 500 typical parameters were sampled from the variance-covariance matrix; 500 random effects were then sampled per population parameter value. The mean response was then calculated per virtual population, with 2.5% and 97.5% percentiles of the generated distribution of the means forming a 95% confidence interval of the true population mean. For model validation, dapagliflozin exposure was sampled directly from NCT02268214 study data. For subsequent forward simulations, the mean values of dapagliflozin AUC or baseline HbA1c were calculated across all studies and used as regressors.
